# Dynamics of the prognostic index during the course of treatment for anaplastic thyroid carcinoma

**DOI:** 10.1007/s00595-024-02991-y

**Published:** 2025-01-06

**Authors:** Masaomi Sen, Ryo Ito, Takeshi Abe, Hiroko Kazusaka, Mami Matsui, Marie Saitou, Ryuta Nagaoka, Tomoo Jikuzono, Iwao Sugitani

**Affiliations:** 1 Department of Endocrine Surgery, Nippon Medical Musashi Kosugi Hospital, 1-383 Kosugi-cho, Nakahara-ku, Kawasaki-shi, Kanagawa 211-8533 Japan; 2 Department of Endocrine Surgery, Nippon Medical School Hospital, 1-1-5 Sendagi, Bunkyo-ku, Tokyo, 113-8603 Japan

**Keywords:** Anaplastic thyroid carcinoma, Prognostic index, Dynamic marker, Dynamics

## Abstract

**Purpose:**

Tumor/node/metastasis staging and prognostic index (PI) are used to predict prognosis and guide treatment for anaplastic thyroid carcinoma (ATC). With the advent of treatments, such as BRAF/MEK inhibitors and immune checkpoint inhibitors, dynamic markers to assess disease status and treatment efficacy are needed. This study examined the utility of PI as a dynamic marker for ATC treatment.

**Methods:**

This retrospective study investigated 20 patients with ATC who were treated aggressively from January 2011 to December 2022. Patients were categorized into low-, high-, low-, and high-high groups according to the PI before and after treatment. Survival was then compared between the groups.

**Results:**

The median overall survival was not reached for the Low–Low, 363 days for the High–Low, 158.5 days for the Low–High, and 90.5 days for the High–High groups (*p* < 0.01). Among the 13 patients with an initially high PI, 6 patients achieved a low PI during treatment, but three showed subsequent increases. Categorizing PI further into decreased, temporarily decreased, and non-decreased groups, median overall survival was 363 days, 158.5 days, and 87 days, respectively (*p* < 0.01).

**Conclusion:**

PI is a critical prognostic indicator that facilitates treatment decision-making for ATC. PI may also have potential as a dynamic marker for assessing the disease status and treatment efficacy.

## Introduction

Anaplastic thyroid carcinoma (ATC) accounts for approximately 1–2% of all thyroid malignancies. This extremely aggressive tumor has a median survival time of 3–6 months post-diagnosis and a 1-year survival rate of 5–20%. No standard treatment has been established for ATC, and patients have been treated using a multimodal approach including surgery, radiation therapy, chemotherapy, and multikinase inhibitors (MKIs). Recent advances in genomic therapy have led to the identification of driver genes in thyroid cancer. The introduction of BRAF/MEK inhibitors targeting the v-raf murine sarcoma viral oncogene homolog B (*BRAF)* V600E mutation and combination treatment with immune checkpoint inhibitors has been reported to improve treatment outcomes [1].

Prognostic index (PI) was devised in 2001 by Sugitani et al. as a tool for predicting the prognosis of patients with ATC. PI includes four adverse prognostic factors: 1) acute symptoms (e.g., hoarseness, dysphagia, dyspnea, rapidly enlarging neck mass) presenting within 1 month, 2) leukocytosis (white blood cell [WBC] count ≥ 10,000/µL), 3) tumor size > 5 cm, and 4) distant metastasis. The PI score is determined by the number of factors present in the patient [2]. Sugitani et al. reported that the 6-month survival rate for patients with a PI of 1 was 62%, whereas no patients with a PI of 3 survived beyond 6 months. Orita et al. prospectively evaluated the efficacy of PI and concluded that it is a valuable tool for predicting prognosis and selecting appropriate treatment strategies for individual patients [3].

In the "Guidelines for the Management of Thyroid Tumors 2018,” prediction of prognosis and treatment decision-making for ATC patients in Japan were based on an algorithm incorporating the tumor/node/metastasis (TNM) staging system and PI [4]. The ,“Guidelines for the Management of Thyroid Tumors 2024” introduced genetic testing for identifying driver genes. However, shared decision-making considering the performance status, TNM staging, and PI remains recommended [5].

Even when ATC is diagnosed at the recurrence of differentiated thyroid carcinoma (DTC), treatment is recommended. Given that PI can be applied not only to common-type ATC, but also to patients with anaplastic transformation in the neck following surgery for DTC [3], we hypothesized that PI could be used not only as an indicator for evaluating the prognosis and determining the treatment strategy at initial diagnosis, but also for monitoring the disease status and evaluating treatment efficacy following therapeutic intervention. This study aimed to clarify the utility of dynamic PI monitoring during the clinical course of ATC.

## Methods

We retrospectively reviewed the medical records of ATC patients who received aggressive treatment between January 2011 and December 2022. Baseline patient characteristics, blood test results, imaging findings, treatment modalities, TNM staging, PI, and survival data were collected for the analysis.

The study included patients ≥ aged 18 years with pathologically confirmed ATC. Twenty total with 20 ATC patients were included in this study. During this 12-year period, we tried to adopt the best available treatment based on TNM staging and PI. The optimal management of ATC is currently radical resection plus multimodal treatment. This study was conducted in accordance with the principles of the Declaration of Helsinki (revised in 2013). The protocol for this retrospective study was approved by the institutional review board of Nippon Medical School Hospital (approval no. B-2023–803), and the requirement for informed consent was waived owing to the retrospective nature of the study.

### Definitions

Cancer staging was determined using the 8th edition of the Union for International Cancer Control (UICC) TNM Classification of Malignant Tumors [6].

Overall survival (OS) was calculated as the duration from the date of pathological diagnosis to the date of death from any cause.

Surgery was defined as a radical resection, excluding tracheostomies alone and biopsies. However, a complete resection is not required.

Multimodal therapy was defined as the administration of two or more treatment modalities including surgery, radiation therapy, chemotherapy, and MKIs.

### Outcomes

#### Age, stage, PI, treatment, and OS

OS was compared according to age, TNM stage, PI, and treatment. A low PI was defined as PI ≤ 1, and a high PI as PI ≥ 2.

#### PI dynamics and OS

We evaluated the association between OS and PI dynamics from the time of diagnosis to the post-treatment evaluation during follow-up. PI dynamics were defined as the change between the initial PI evaluated before treatment (pre-treatment) and the PI re-evaluated at the completion of all treatments (post-treatment). However, for some patients who were alive after the completion of treatment, the PI at the most recent point in the follow-up period was used as the post-treatment PI. The following are the differences in the evaluation methods between post-treatment PI and pre-treatment PI. Acute symptoms after treatment were defined as recurrence and/or distant metastasis within 1 month after treatment, progression of residual lesions, deterioration of general condition due to cancer progression, or appearance of new symptoms excluding postoperative recurrent laryngeal nerve palsy. The maximum diameter of the primary tumor was measured in patients who had not undergone surgery, and the maximum diameter of the recurrent or metastatic tumor was measured in patients who had undergone surgery. The assessment of leukocytosis and distant metastasis was the same as for pre-treatment PI. Patients were classified into four groups based on PI dynamics, and OS was compared between the groups. The high–high (HH) group was defined as patients who maintained a high PI from the time of diagnosis, the high–low (HL) group was defined as patients who decreased from high PI to low PI, the low–high (LH) group as patients who increased from low PI to high PI, and the low–low (LL) group as patients who maintained a low PI. In addition, the initial PI, minimum PI during the course of treatment, and PI at the end of follow-up were measured in patients with a high PI in ATC. Each PI was re-evaluated at the most recent time point from the end of each treatment. However, postoperative PI was evaluated at a median of 31.5 days (range, 12–60 days). We defined 1st PI as measured after primary treatment, 2nd PI as measured after secondary treatment, 3rd PI as measured after tertiary treatment, 4th PI as measured after quaternary treatment, and the final PI as measured at the end of the follow-up period, including 4th PI. Patients with an initial PI of high were also categorized into the high–low–low group (decreased PI group), the high–low–low group (temporarily decreased group), and the high–high–high group (non-decreased group), and OS was compared among these three subgroups.

### Statistical analysis

Statistical analyses were performed using the EZR software program [7]. Kaplan–Meier curves were generated, and differences in OS were examined using the log-rank test, with values of p < 0.05 considered statistically significant. Outcomes by PI dynamics were compared using four groups (HH, LH, LH, and LL) and three subgroups (decreased PI group, temporarily decreased group, and non-decreased group). Due to the small sample sizes, comparisons could not be made for each group or subgroup.

## Results

### Background characteristics

Table 1 shows the characteristics of the 20 patients, comprising 15 women and five men. The median age of the patients was 77 years (range, 60–87 years). Twelve patients had Stage IVB disease, and the remaining eight patients had Stage IVC disease. The initial PI was low in 7 patients and high in 13. Fifteen patients underwent surgery and almost half (11 patients) received multimodal treatment. The outcome was death in 14 patients, and the median OS was 145 days (range, 18–3261 days). All the radiation therapies were administered postoperatively. Paclitaxel and lenvatinib were administered for chemotherapy and MKI, respectively (Table 1).

**Table 1:** Patient characteristics

		*N =* 20
Age, y		77 (60–87)
Sex	Female / Male	15 (75%) / 5 (25%)
Stage	IVA / IVB / IVC	0 (0%) / 12 (60%) / 8 (40%)
Prognostic Index (PI)	Low (0/1)	3 (15%) / 4 (20%)
	High (2/3/4)	6 (30%) / 4 (20%) / 3 (15%)
Treatment		
Surgery		16 (80%)
R0 / R1 / R2 or palliative surgery		7 (43.8%) / 7 (43.8%) / 2 (12.5%)
Radiation therapy		5 (25%)
Chemotherapy (paclitaxel)		7 (35%)
Multikinase inhibitor (lenvatinib)		7 (35%)
Multidisciplinary treatment (use of ≥ 2 of the above)		11 (55%)
Outcome	Alive	6 (30%)
	Dead	14 (70%)
Overall survival (days)		145 (18–3261)

Values are given as the number (percentage) or median (range)

#### Age, stage, PI, treatment, and OS

The median OS was 177 days (range, 45–1790 days) for Stage IVB and 127 days (range, 18–3261 days) for Stage IVC (*p* = 0.64). The median OS was not reached (range, 140–1790 days) for low PI (PI ≤ 1) and 104 days (range, 18–3621 days) for high PI (PI ≥ 2) (*p* < 0.05).

According to the treatment method, the median OS was 253 days (range: 48–3261 days) for surgical cases and 80 days (range: 18–140 days) for nonsurgical cases (*p* < 0.01). The median OS was not reached (range: 71–3261 days) for multidisciplinary treatment and 104 days (range: 18–363 days) for monotherapy. No significant difference in OS was observed between the groups for radiation therapy, chemotherapy, or MKIs (Table 2).

**Table 2:** Stage, PI, treatment method, and overall survival

		*n*	OS, days	*p*-value (log-rank)
Stage	IVB	12	177 (45–1790)	0.64
	IVC	8	127 (18–3261)	
PI	Low PI	7	NA (140–1790)	< 0.05
	High PI	13	104 (18–3261)	
Surgery	Present	16	253 (48–3261)	< 0.01
	Absent	4	80 (18–140)	
Radiation therapy	Present	5	NA (71–1790)	0.12
	Absent	15	140 (18–3261)	
Chemotherapy (paclitaxel)	Present	7	177 (45–3261)	0.83
	Absent	13	150 (18–1790)	
Multikinase inhibitor (lenvatinib)	Present	7	150 (18–1043)	0.4
	Absent	13	363 (45–3261)	
Multidisciplinary treatment	Present	11	NA (71–3261)	< 0.01
(use of ≥ 2 of the above)	Absent	9	104 (18–363)	

Values for OS are given as the median (range)

*PI* Prognostic index, *OS* overall survival, *NA* Not available

#### PI dynamics and OS

Table 3 shows the treatment and the initial and post-treatment PI dynamics of the 20 patients. Of the 14 patients who died, 12 had local recurrence or distant metastasis due to ATC, and 2 had pneumonia (Cases 10 and 16).

**Table 3:** Characteristics of the 20 patients with anaplastic thyroid carcinoma who received aggressive treatment

No	Age (y)	Sex	Stage	Initial PI	Post-treatment PI	Treatment	OS (days)	Outcome
1	80	M	IVC	4	1	S(R1) + C	3261	Alive
2	63	F	IVB	2	4	C	115	Dead
3	87	F	IVC	3	3	S(R1)	94	Dead
4	82	F	IVB	1	4	S(R1) + RT + C + MKI	177	Dead
5	87	M	IVB	1	3	C + MKI	140	Dead
6	78	F	IVC	4	4	MKI	18	Dead
7	74	F	IVC	3	3	S(R2) + MKI	150	Dead
8	79	F	IVB	2	0	S(R1)	363	Dead
9	66	F	IVC	3	2	S(R1)	104	Dead
10	75	M	IVB	0	0	S(R1)	513	Dead
11	72	F	IVC	2	2	S(R0) + MKI	87	Dead
12	82	F	IVB	0	0	S(R0) + RT	1790	Alive
13	60	M	IVC	4	4	C + S(R0) + C + MKI	253	Dead
14	68	F	IVB	0	0	S(R0) + RT	1588	Alive
15	76	F	IVC	1	1	S(R0) + MKI	1043	Alive
16	79	M	IVB	2	2	C	45	Dead
17	73	F	IVB	2	3	S(R1) + RT	71	Dead
18	85	F	IVB	3	2	S(R2)	48	Dead
19	68	F	IVB	2	1	S(R0) + MKI	119	Alive
20	85	F	IVB	1	0	S(R0) + RT	748	Alive

*S* surgery, *RT* radiation therapy, *C* chemotherapy, *MKI* multikinase inhibitor, *(R)* resectability, *PI* prognostic index, *OS* overall survival, *M* male, *F* female

**Fig. 1:**
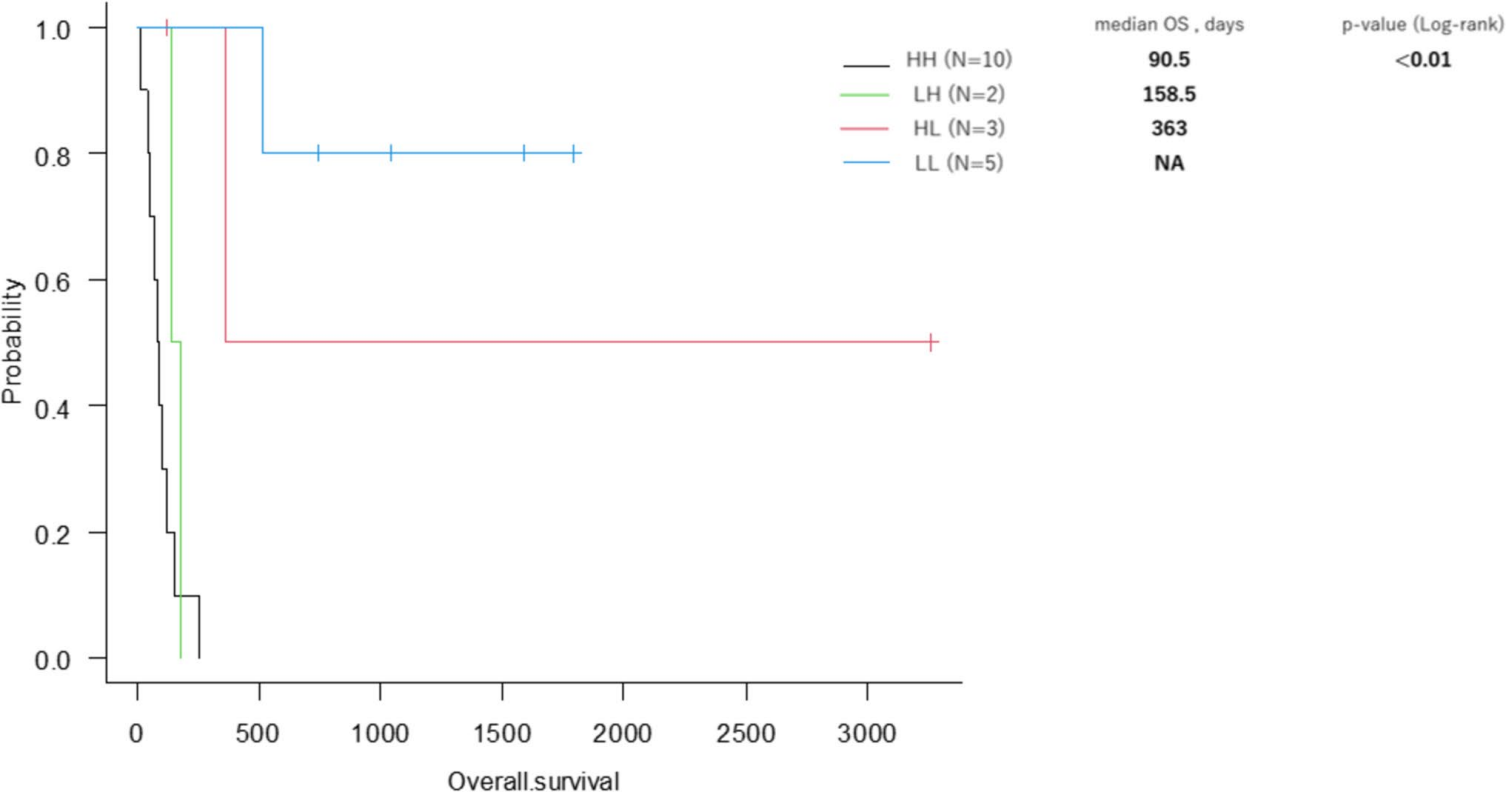
Overall survival for patients in the four groups based on the PI dynamics. The median OS was NA (range: 513–1790 days) in the LL group (*n =* 5), 363 days (range: 119–3261 days) in the HL group (*n =* 3), 158.5 days (range: 140–177 days) in the LH group (*n =* 2), and 90.5 days (range: 18–253 days) in the HH group (*n =* 10) (*p* < 0.01)

The median OS was not reached (range: 513–1790 days) in the LL group (*n* = 5), 363 days (range: 119–3261 days) in the HL group (*n* = 3), 158.5 days (range: 140–177 days) in the LH group (*n* = 2) and 90.5 days (range: 18–253 days) in the HH group (*n* = 10) (*p* < 0.01) (Fig. 1).

Table 4 shows the characteristics of ATC patients (10 women and 3 men) with a high initial PI. Patients ranged in age from 60 to 87 years, and the disease stage was IVB in six patients and IVC in seven. Surgical treatment was performed in 10 patients, of whom 6 achieved a low PI postoperatively. Multidisciplinary treatment was performed in seven patients, of whom only three survived longer than 6 months. The decrease in PI was influenced by improvements in symptoms (*n =* 5), reduction in tumor size (*n =* 6), and decrease in WBC count (*n =* 3). Unfortunately, a re-elevation of PI was observed in three cases, attributed to a progression of residual disease (*n =* 1), a re-elevation of the WBC count (*n =* 1), and the appearance of distant metastases (*n =* 1).

**Table 4:** Changes in PI during the course of treatment for high PI cases

No	Age (y)	Sex	Stage	Initial PI	1st PI	2nd PI	3rd PI	4th PI	Final PI	Treatment	OS (days)	Outcome
1	80	M	IVC	4	1	1			1	S + C	3261	Alive
2	79	F	IVB	2	0				0	S + MKI	363	Dead
3	60	M	IVC	4	3	1	2	4	4	C + S + C + MKI	253	Dead
4	74	F	IVC	3	1	3			3	S + MKI	150	Dead
5	68	F	IVB	2	0	1			1	S + C	119	Alive
6	63	F	IVB	2	4				4	C	115	Dead
7	66	F	IVC	3	2				2	S	104	Dead
8	87	F	IVC	3	3				4	S	94	Dead
9	72	F	IVC	2	2	2			2	S + MKI	87	Dead
10	73	F	IVB	2	2	3			3	S + RT	71	Dead
11	85	F	IVB	3	1				2	S	48	Dead
12	79	M	IVB	2	2				2	C	45	Dead
13	78	F	IVC	4	4				4	MKI	18	Dead

*S* surgery, *RT* radiation therapy, *C* chemotherapy, *MKI* multikinase inhibitor, *PI* prognostic index, *OS* overall survival, *M* male, *F* female

Among the 7 patients for whom PI did not decrease, the main causes were the presence of distant metastases (*n =* 2), persistent leukocytosis (*n =* 3), short-term recurrence or distant metastases (*n =* 2), and progression of residual disease (*n =* 1).

**Fig. 2:**
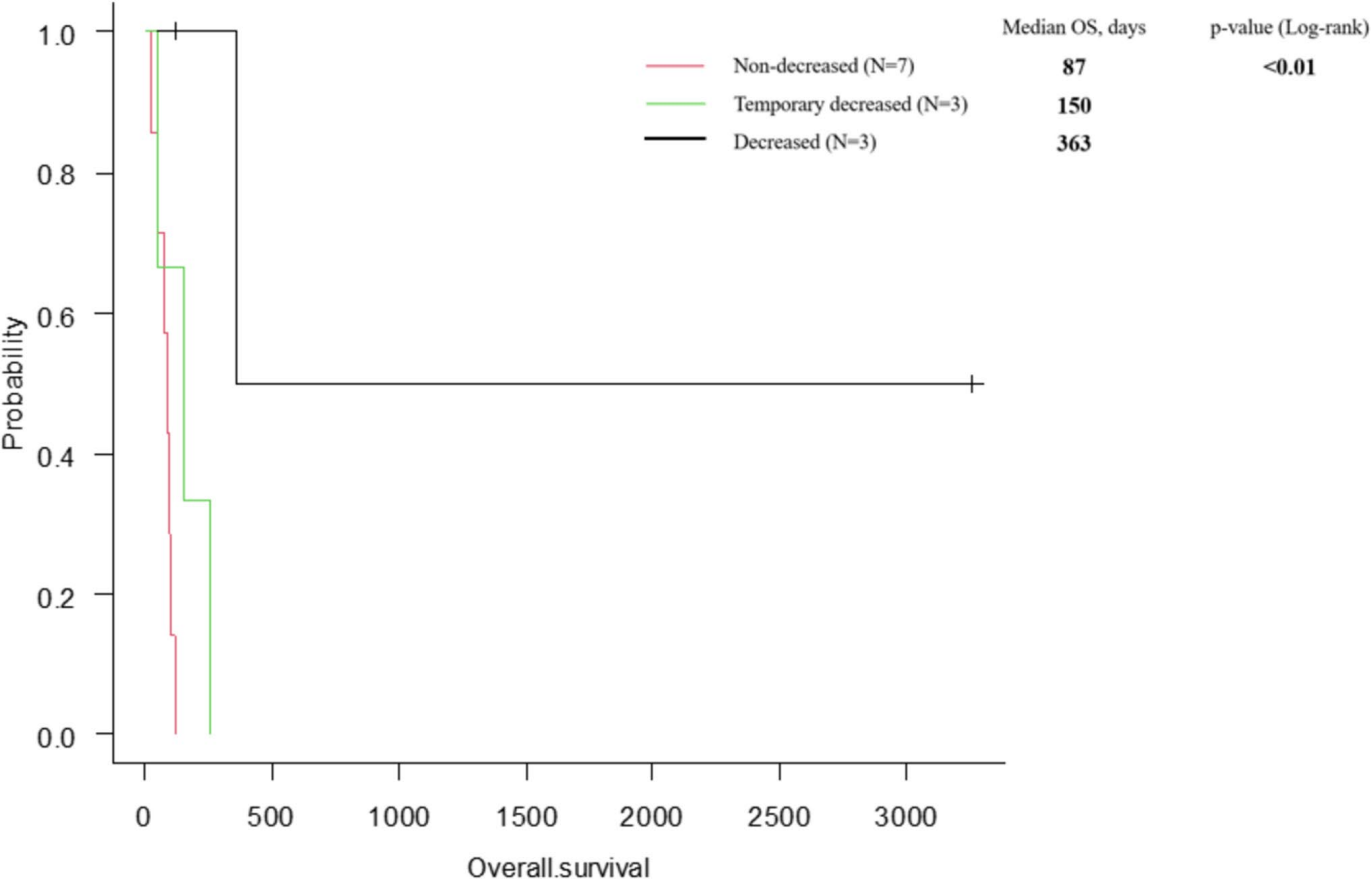
Overall survival for the PI dynamics in high PI patients during the course of treatment. The median OS was 363 days (range: 119–3261 days) in the decreased group (*n =* 3), 150 days (range: 140–177 days) in the temporarily decreased group (*n =* 3), and 87.5 days (range: 18–254 days) in the non-decreased group (*n =* 7) (*p* < 0.01)

The median OS for the 13 patients with a high pre-treatment PI was 363 days (range: 119–3261 days) in the decreased group (*n =* 3), 150 days (range: 140–177 days) in the temporarily decreased group (*n =* 3), and 87.5 days (range: 18–254 days) in the non-decreased group (*n =* 7) (*p* < 0.01) (Fig. 2).

## Discussion

PI can be assessed before starting treatment based on the number of applicable items among four different poor prognostic factors and reflects survival prognosis. This indicator is extremely important for determining the treatment strategies for ATC. Several reports have estimated the effectiveness of treatment based on PI. Sugitani et al. reported that the prognosis for Stage IVB (according to UICC 7th edition [8]) was similar for cases treated with a radical resection with or without an extended resection and better than for cases treated with debulking surgery or non-operatively. However, the 1-year survival rate of patients with PI ≤ 1 was 50% compared to 11% for patients with PI ≥ 2, thus indicating that PI is useful in determining the indications for an extended radical resection [9]. Among ATC patients who received paclitaxel as neoadjuvant chemotherapy, PI ≥ 2 was found to be an independent prognostic factor, but it was not associated with a response to paclitaxel [10]. Among the ATC patients treated with MKIs, those with a low PI (PI ≤ 1) showed a longer survival (17.4 months) than those with high PI (PI ≥ 2; 3.8 months, *p* = 0.37), thus suggesting the potential usefulness of PI as a predictor of the effectiveness of MKI treatment [11]. PI has been suggested to be useful not only for the prediction of the individual prognosis and treatment decision-making, but also for estimating the efficacy of each treatment.

The neutrophil-to-lymphocyte ratio (NLR) has been reported to be a strong prognostic factor in cancers of various organs. This ratio has attracted attention as a dynamic prognostic marker of ATC. Among patients with ATC, the median OS was significantly worse in the increased NLR group (7.7 months) than in the non-increased group (23.5 months; *p* < 0.001) [12]. The median OS in the increased NLR group was 2.0 months after starting lenvatinib treatment, and the median OS in the non-increased group was 5.3 months (*p* = 0.003) [13]. Ishihara et al. reported that a reduction in NLR during MKI treatment may indicate a sustained drug efficacy [11]. Patients with a reduction in NLR from baseline on day 14 after starting lenvatinib treatment showed a slightly but significantly higher overall response rate (42.9%) than those who did not (0%, *p* = 0.19) [14]. The NLR is an important prognostic score reflecting systemic inflammation due to cancer progression; however, it is only a single factor.

This study investigated the relationship between the PI dynamics and OS in ATC patients. Unlike the NLR, the PI is composed of four predictors. In addition, unlike TNM, PI is applicable not only to conventional ATC, but also to anaplastic transformation in the neck following surgery for DTC [3]. Taking advantage of these characteristics, we hypothesized that PI can be used as a dynamic marker to evaluate disease progression during the course of treatment and determine treatment efficacy, thus taking advantage of the availability of this index for any type of ATC.

In the dynamics of PI from the time of diagnosis to follow-up, cases with decreased PI and cases with maintenance of low PI achieved significantly longer OS than cases with increased PI or maintenance of high PI, thus suggesting that a reevaluation of PI during the disease course may be useful. This study found a significant difference in OS between patients with high and low initial PIs and a significant difference in OS between patients with decreased and non-decreased PI due to therapeutic intervention, thus suggesting the benefit of reevaluating PI during the course of treatment.

The dynamics of the PI reflected the treatment effects. Surgery contributed to a decrease in PI in patients with ATC, and OS was significantly longer in surgical cases than in nonsurgical cases. In contrast, radiation therapy, chemotherapy, and MKI maintained the PI, but did not decrease it. This is consistent with the present findings, which showed that while multidisciplinary therapy correlated significantly with OS, no such correlation was observed with monotherapy, excluding surgery. The next treatment other than surgery should therefore be considered before PI reaches ≥ 2.

Recent advances in genomic therapy have led to the introduction of BRAF/MEK inhibitors. In The Rare Oncology Agnostic Research basket study, dabrafenib + trametinib treatment in patients with ATC showed good efficacy and a good safety profile [15, 16]. The efficacy of the combination of dabrafenib and trametinib (+ immune checkpoint inhibitors) as a preoperative drug therapy has been reported [17]. In ATC, for which no standard treatment has been determined, the advent of BRAF/MEK inhibitors (and their combination with immune checkpoint inhibitors) has been a game changer, and improvements in the treatment outcomes have been reported [1]. As the prognosis of ATC continues to improve, the precise assessment of treatment with dynamic markers is required.

This study was associated with several limitations. First, this was a single-center retrospective study with a small sample size and selection bias owing to the exclusion of patients with ATC who were not receiving aggressive treatment or had not been histopathologically diagnosed. Second, the PI was measured at various times, including at diagnosis, postoperatively, at the end of radiotherapy, at the end of chemotherapy, and at the end of MKI administration. Finally, although each patient was treated with the best available therapy at the time, different treatment strategies may have affected OS and the PI dynamics.

In conclusion, PI monitoring may provide a more accurate estimate of the ATC prognosis during treatment. Further large prospective studies are needed to clarify the association between PI monitoring and the outcomes in patients with ATC.

## Data Availability

The authors declare that all data related to this article are available in this manuscript.

## References

[1] Maniakas A, Dadu R, Busaidy NL, Wang JR, Ferrarotto R, Lu C, et al. Evaluation of overall survival in patients with anaplastic thyroid carcinoma. JAMA Oncol. 2020;6:1397–404. 32761153 10.1001/jamaoncol.2020.3362 PMC7411939

[2] Sugitani I, Kasai N, Fujimoto Y, Yanagisawa A. Prognostic factors and therapeutic strategy for anaplastic carcinoma of the thyroid. World J Surg. 2001;25:617–22. 11369989 10.1007/s002680020166

[3] Orita Y, Sugitani I, Amemiya T, Fujimoto Y. Prospective application of our novel prognostic index in the treatment of anaplastic thyroid carcinoma. Surgery. 2011;150:1212–9. 22136842 10.1016/j.surg.2011.09.005

[4] The JAES/JSTS Task Force on the Guidelines for Thyroid Tumors. Clinical practice guidelines on the management of thyroid tumors 2018. Nihon Naibunpitu Kojosen Geka Gakkai Zasshi. 2018;35(Suppl 3):1–87.

[5] The JAES Task Force on the Guidelines for Thyroid Tumors. Clinical practice guidelines on the management of thyroid tumors 2024. Nihon naibunpitu geka Gakkai zasshi. 2024;41(Suppl 2):1–116.

[6] Brierly JD, Gospodarowics MK, Wittekind C, Union for International Cancer Control (UICC), et al. TNM classification of malignant tumours. 8th ed. Hoboken, NJ: Wiley; 2017. p. 256.

[7] Kanda Y. Investigation of the freely available easy-to-use software ‘EZR’ for medical statistics. Bone Marrow Transplant. 2013;48:452–8. 23208313 10.1038/bmt.2012.244 PMC3590441

[8] Sobin LH, Gospodarowicz MK, Wittekind C. TNM classification of malignant tumours. 7th ed. New York: Wiley; 2009.

[9] Sugitani I, Hasegawa Y, Sugasawa M, Tori M, Higashiyama T, Miyazaki M, et al. Super-radical surgery for anaplastic thyroid carcinoma: a large cohort study using the Anaplastic Thyroid Carcinoma Research Consortium of Japan database. Head Neck. 2014;36:328–33. 23729360 10.1002/hed.23295

[10] Yamazaki H, Sugino K, Katoh R, Matsuzu K, Masaki C, Akaishi J, et al. Response to neoadjuvant paclitaxel predicts survival in anaplastic thyroid carcinoma. Cancer Med. 2023;12:3027–35. 36052510 10.1002/cam4.5219 PMC9939216

[11] Ishihara S, Onoda N, Noda S, Tauchi Y, Morisaki T, Asano Y, et al. Treatment of anaplastic thyroid cancer with tyrosine kinase inhibitors targeted on the tumor vasculature: initial experience in clinical practice. Endocr J. 2021;68:63–8. 32863283 10.1507/endocrj.EJ20-0287

[12] Yamazaki H, Sugino K, Matsuzu K, Masaki C, Akaishi J, Hames K, et al. Inflammatory biomarkers and dynamics of neutrophil-to-lymphocyte ratio in anaplastic thyroid carcinoma. Endocrine. 2020;70:115–22. 32307657 10.1007/s12020-020-02313-5

[13] Yamazaki H, Iwasaki H, Suganuma N, Toda S, Masudo K, Nakayama H, et al. Inflammatory biomarkers and dynamics of neutrophil-to-lymphocyte ratio in lenvatinib treatment for anaplastic thyroid carcinoma. Gland Surg. 2021;10:852–60. 33842230 10.21037/gs-20-871 PMC8033089

[14] Fukuda N, Toda K, Fujiwara YU, Wang X, Ohmoto A, Urasaki T, et al. Neutrophil-to-Lymphocyte ratio as a prognostic marker for anaplastic thyroid cancer treated with Lenvatinib. In Vivo. 2020;34:2859–64. 32871825 10.21873/invivo.12113 PMC7652499

[15] Subbiah V, Kreitman RJ, Wainberg ZA, Cho JY, Schellens JHM, Soria JC, et al. Dabrafenib and trametinib treatment in patients with locally advanced or metastatic BRAF V600-mutant anaplastic thyroid cancer. J Clin Oncol. 2018;36:7–13. 29072975 10.1200/JCO.2017.73.6785 PMC5791845

[16] Subbiah V, Kreitman RJ, Wainberg ZA, Cho JY, Schellens JHM, Soria JC, et al. Dabrafenib plus trametinib in patients with BRAF V600E-mutant anaplastic thyroid cancer: updated analysis from the phase II ROAR basket study. Ann Oncol. 2022;33:406–15. 35026411 10.1016/j.annonc.2021.12.014 PMC9338780

[17] Zhao X, Wang JR, Dadu R, Busaidy NL, Xu L, Learned KO, et al. Surgery after BRAF-directed therapy is associated with improved survival in BRAF. Thyroid. 2023;33:484–91. 36762947 10.1089/thy.2022.0504 PMC10122263

